# Molecular encapsulation of andrographolide in 2-hydroxypropyl-*β*-cyclodextrin cavity: synthesis, characterization, pharmacokinetic and *in vitro* antiviral activity analysis against SARS-CoV-2

**DOI:** 10.1016/j.heliyon.2021.e07741

**Published:** 2021-08-09

**Authors:** Shashi Chandrama Singh, Dharmendra Kumar Khatri, Kulbhaskar Singh, Vinay Kumar Kanchupalli, Jitender Madan, Shashi Bala Singh, Harshpal Singh

**Affiliations:** a Research and Development Centre, Ambe Phytoextracts Private Limited, Pauri Garhwal, Uttarakhand, India; b Department of Biological Sciences, National Institute of Pharmaceutical Education and Research, Hyderabad, India; c Department of Chemical Sciences, National Institute of Pharmaceutical Education and Research, Hyderabad, India; d Department of Pharmaceutics, National Institute of Pharmaceutical Education and Research, Hyderabad, India

**Keywords:** Andrographolide, Cyclodextrin, SARS-CoV-2, Pharmacokinetic, Bioavailability, Antiviral

## Abstract

In present investigation, AND-2-HyP-*β*-CYD (Andrographolide-2-Hydroxypropyl-*β*-cyclodextrin) complex was synthesized and characterized for antiviral and pharmacokinetic profile. The linear host-guest relation suggested synthesis of a 1:1 complex of AND with 2-HyP-*β*-CYD by inclusion mode. The Kc, stability constant of the two phase system of AND with 2-HyP-*β*-CYD computed to be 38.60 x 10^−3^M. ^1^H NMR spectrum of AND indicated the presence of triplet at 6.63-ppm which was up-fielded in AND-2-HyP-*β*-CYD complex at 6.60-ppm (doublet) confirmed the insertion of AND in cavity of 2-HyP-*β*-CYD through lactone ring. AND-2-HyP-β-CYD complex exhibited the IC_50_ of 0.1-*μ*g.mL^−1^ (E gene) and 0.29-*μ*g.mL^−1^ (N gene) against SARS-CoV-2 infected Vero6 cells. Moreover, a 1.5-fold increment in extent of absorption of AND was noticed post complexation. The bioavailability was estimated to be 15.87 ± 3.84% and 23.84 ± 5.46%, respectively for AND and AND-2-HyP-*β*-CYD complex. AND-2-HyP-*β*-CYD complex may be a prospective candidate for further studies to evolve as a clinically viable formulation against SARS-CoV-2.

## 1 Introduction

*Andrographis paniculata* (*A. Paniculata*, Family: *Acanthaceae*), a medicinal herb, is also best-known as Kalmegh/Bhunimba/Chiraita [1]. Moreover, A. *Paniculata* is also used in detoxification and getting rid of heat. Hence, it is also considered as cold property herb in Traditional Chinese Medicines [2]. Fresh leaf juice extract of A. *paniculata* has also been reported for the treatment of muscle contraction pain, poor appetite, casual stools and diarrhea [3] in addition to gastrointestinal canal and infections of upper respiratory tract.

Andrographolide (AND), a diterpenoid lactone, is a chief therapeutically active moiety present in aerial parts of *A. Paniculata* [4]. Furthermore, AND is also responsible for the bitter taste of *A. Paniculata* and thus, it has been also referred to as ‘*King of Bitters*’.

Recently, SARS-CoV-2 (Severe Acute Respiratory Syndrome-Coronavirus-2) virus has caused a pandemic COVID-19 that includes symptoms like pyrexia, tussis, tiredness, dyspnea as well as failure to smell or taste [5]. AND molecule displayed high affinity for SARS-CoV-2 virus protease with -3.094357 *K*cal/mol as docking score [6]. H-bond and salt bridge interactions were noticed to be involved in all the binding conformations of AND in the active binding pocket. Mechanistically, AND with 4-H bonds using 3 motifs, namely Gly143, Cys145 and Glu166 bound to protease. Hence, delivery of a high concentration of AND in the vicinity of SARS-CoV-2 pocket is mandatory to exhibit the therapeutic effect.

Pharmacokinetic study displayed 9.27 ± 1.69% as absolute oral bioavailability of AND in addition to C_max_ of 0.73 ± 0.17 *μ*M.L^−1^ and T_max_ of 0.42 ± 0.14 h. AND exhibited rapid plasma clearance with half-lives (*t*_1/2_) of 1.86 ± 0.21 h and 3.30 ± 0.35 h following *i.v. (intravenous)* and oral administration, respectively [7]. The poor oral bioavailability of AND may be credited to physicochemical as well as biopharmaceutical factors such as low water solubility (3.29 ± 0.73 *μ*g.mL^−1^) [8] and high hydrophobicity due to *log P* of 2.632 ± 0.135 [9]. In addition, rapid biotransformation and efflux by P-gp (Pumping glycoprotein) also reduce the oral bioavailability of AND [8]. About 55% of the AND binds with human plasma proteins [10].

2- Hydroxypropyl-β-cyclodextrin, 2-HyP-*β*-CYD is an analogue of cyclodextrin (CYD), a macrocycle composed of seven *α*-D-glucopyranoside units. The US-FDA (Food and Drug Administration) listed 2-HyP-*β*-CYD (Aqueous solubility>500 mg.mL^−1^ at room temperature) as approved inert material for oral and intravenous administration [11]. Moreover, 2-HyP-*β*-CYD has been widely employed for improving biopharmaceutical chattels of a number of pharmaceutical products including antihypertensive drug [12], antimicrobial drugs [13, 14], and antiviral drugs through both oral and inhalation route of administration [15, 16].

Therefore, in the present investigation, AND-2-HyP-*β*-CYD complex was formulated both in solid and solution state and characterized under a set of rigorous *in vitro* as well as *in vivo* parameters to exhibit its therapeutic prospective against SARS-CoV-2 virus.

## 2 Materials and methods

### 2.1 Materials

Andrographolide (AND, 95% w/w, Mw~350.45 Da) as well as 2-Hydroxypropyl-β-cyclodextrin (2-HyP-*β*-CYD) were supplied by in-house facility of M/s Ambe Phytoextracts Private Limited, Pauri Garhwal, Uttarakhand, India. Methanol and water of higher purity (HPLC grade) were procured from Loba Chemie, (Mumbai, Maharashtra) India. All other reagents were of higher analytical level.

### 2.2 Phase solubility assay

The experiment of phase solubility was performed to compute the ratio of the AND-2-HyP-*β*-CYD-complex in the solution phase [17]. In brief, AND (10 mg) was dispersed separately in aqueous phase (10 mL) containing 2–8 mM of powder of 2-HyP-*β*-CYD. All the samples were then kept in an orbital shaker for constant stirring at 100 rpm for 5 successive days at 37 ± 2 °C. The AND suspension samples were passed individually through a 0.22-*μ*m membrane filter and examined for drug content by HPLC method [18]. The *K*c, apparent stability constant [17] was computed from the slope of the phase-solubility curve using Eq. (1):

[1] Kc=Slope/[So∗(1−Slope)]

S_o_ depicts the solubility of AND in the absence of 2-HyP-*β*-CYD.

### 2.3 HPLC instrumentation and analysis of andrographolide

HPLC Instrument (Waters e2695) equipped with C8 column (4.6 × 2.5 cm, 5 *μ*m) was used for the analysis of AND. Stock solution of AND was prepared by dissolving the compound in methanol. Further, diluent (50% methanol and 50% water) was employed to get the concentration range of 10–70-*μ*g/mL of AND. The mobile phase was formulated with 60% methanol and 40% water. The detection was carried out at absorption maxima of 225 nm [18]. All observations were noticed in triplicate (n = 3).

### 2.4 Synthesis and characterization of solid complex

The complex of AND with 2-HyP-*β*-CYD in solid phase was fabricated by optimized cost effective solvent evaporation method [19]. In brief, AND (35 mg, 5 mM) and 2-HyP-*β*-CYD (146 mg, 5 mM) were solubilized in absolute ethanol (20 mL) with vigorous stirring for 2 h at room temperature. Subsequently, absolute ethanol was evaporated using rotary flash evaporator at 50 °C and finally, placed in a dessicator for complete drying of the complex, AND-2-HyP-*β*-CYD.

### 2.5 Characterization of solid complex

#### 2.5.1 ATR (attenuated total reflectance)

The initial characterization of solid complex, AND-2-HyP-*β*-CYD was carried out using ATR spectroscopy (Parkin Elmer). The spectrum of AND, 2-HyP-*β*-CYD, physical mixture (AND with 2-HyP-*β*-CYD, 1:1 mM) as well as AND-2-HyP-*β*-CYD (1:1 mM) complex was recorded (resolution of 4 cm^−1^) in the range of 4000–400 cm^−1^.

#### 2.5.2 ^1^H NMR and ROESY (nuclear magnetic resonance and rotating frame overhause effect spectroscopy)

The molecular interactions within AND-2-HyP-*β*-CYD complex were deciphered using ^1^H NMR spectroscopy (BRUKER DPX 500 MHz spectrometer). The deuterated dimethyl sulfoxide (DMSO-d_6_) was used to prepare the solution of AND while 2-HyP-*β*-CYD and AND-2-HyP-*β*-CYD complex were incorporated in deuterated water (D_2_O), separately. All the samples were then poured in NMR tubes. The 2D ROESY spectrum with a sweep width of 8.5 ppm in both dimensions and mixing time of 150 ms, was captured for AND-2-HyP-*β*-CYD complex. The spectrum was developed with 32 scans in addition to 2048 data points in t_2_ and 512 free induced decays (FIDs) in t_1_.

#### 2.5.3 In silico docking analysis

The AND structure was designed using Schrodinger suite 2017-4 with 2 D sketcher (LigPrep, Schrödinger, New York, 2017). By using LigPrep module, the chemical structure was optimized for docking by assigning the bond orders and angles [20]. The 2D structure of AND was transformed into the 3D structure in LigPrep module, and the energy was lessened using OPLS3 [21]. The AND (ionization state) was created at pH 7.0 ± 2.0 with the help of Epik module of LigPrep while rest of the parameters were set to default values. The 3D coordinates of 2-HyP-*β*-CYD were isolated from the X-ray crystal structure of CD glycosyltransferase from Protein Data Bank (rcsb.org) with PDB ID: 3CGT. The assessment and refinement of protein structure were done using protein preparation wizard (GLIDE) [20, 22]. The structure of the protein was minimized and then 2-HyP-*β*-CYD was extracted and minimized. The minimized coordinates of 2-HyP-*β*-CYD were used for the further steps. The grid for the binding site of 2-HyP-*β*-CYD was created by selecting the centroid of the 2-HyP-*β*-CYD. Molecular docking of AND with 2-HyP-*β*-CYD was performed using GLIDE docking module from the Schrödinger Suite. Extra precision (XP) docking calculations were performed and the constants of scaling factor and partial charge cut-off were fixed at the default values of 0.80 and 0.15, respectively [23].

#### 2.5.4 DSC (differential scanning calorimetry)

The synthesis of complex in solid state was also ascertained by employing Differential Scanning Calorimeter (DSC-7, Mettler Toledo). The thermogram of AND, 2-HyP-*β*-CYD, physical mixture of AND with 2-HyP-*β*-CYD (1:1) and AND-2-HyP-*β*-CYD complex was captured at 10 °C/min (heating rate) over temperature limit of 30–260 °C under the purgation of nitrogen.

#### 2.5.5 PXRD (powder X-ray diffraction)

PXRD (Rigaku Corp., Japan) was employed to determine the crystalline architecture of solid complex at the scanning rate of 1°/min over 0–60° diffraction angle (2*θ*) range. PXRD pattern of AND, 2-HyP-*β*-CYD, physical mixture (AND and 2-HyP-*β*-CYD, 1:1 mM) and AND-2-HyP-*β*-CYD complex was attained using Rotaflex, RV 200 powder XRD using Ni-filtered, Cu Ka-radiation, with current of 40 mA and a voltage of 40 kV.

#### 2.5.6 SEM (scanning electron microscopy)

The morphology of solid inclusion complex was analyzed by employing SEM (Jeol/JEM 2100). Sample of AND, 2-HyP-*β*-CYD, physical mixture (AND with 2-HyP-*β*-CYD, 1:1 mM) and AND-2-HyP-*β*-CYD complex was analyzed by forming a thin film on an aluminum stub. The stubs were then covered to a thickness of 200–500 *Å* of gold under an argon atmosphere. The coated samples were then examined and photographs were taken.

#### 2.5.7 Assay of synthesized solid complex

The assay of solid complex was carried out using HPLC method [18]. In brief, 50 mg of AND-2-HyP-*β*-CYD complex was suspended in 100 mL of methanol. Subsequently 1 mL of the sample was diluted to 10 mL with diluent (50%:50%, water: methanol) and filtered through 0.22-*μ*m membrane filter. The sample was assayed for drug content at 225 nm. All observations were noticed in triplicate (n = 3) and % assay [17] was calculated using Eq. (2):

[2] $$\text{ \% Assay}=\frac{\text{Amount of AND recovered}}{\text{Amount of AND}-\text{2}-\text{HyP}-\beta -\text{CYD complex added}}\times 100$$

#### 2.5.8 Solubility analysis

The solubility of solid complex was measured by saturated solution method [14]. In brief, 30 mg of AND-2-HyP-*β*-CYD complex was suspended in 10 mL of aqueous phase and kept at 37±1 °C in an orbital incubator shaker for 24 h at 100 rpm. Subsequently, suspension was filtered and 0.1 mL was diluted to 1 mL with water. The contents in the aliquot were measured by HPLC method [18]. All measurements were noticed in triplicate (n = 3).

#### 2.5.9 Dissolution test analysis

Dissolution testing was carried out using USP dissolution rate test apparatus as per the standard protocol with HPLC method [18] as specified earlier. In brief, 100 mg of AND or 100 mg of AND equivalent in AND-2-HyP-*β*-CYD complex powder was suspended in simulated intestinal fluid (SIF, pH 7.2, 900 mL) and paddle was rotated at 50 rpm. The temperature of SIF was kept at 37 ± 2 °C. At specified time intervals, 2 mL of the SIF was taken and replaced with identical volume. Subsequently, all the samples were passed separately through 0.22 *μ*m membrane filter and assayed for drug content at 225 nm.

### 2.6 *In vitro* antiviral activity analysis

#### 2.6.1 Cytotoxicity testing using standard cell proliferation assay

The cytotoxic effect of AND-2-HyP-*β*-CYD complex on the property of Vero E6 cells was evaluated by using MTT dye [3-(4,5-dimethylthiazol-2-yl)-2,5-diphenyltetrazolium bromide] assay technique [24]. In brief, 1 × 10^4^ Vero E6 cells were incubated for 24 h in serum DMEM (Dulbecco's Modified Eagle Medium) of about 200-*μ*l in a 96 wells microtiter plate. Subsequently, Vero E6 cells were incubated for 24 h as well as 72 h with 10 *μ*g.mL^−1^ or 100-*μ*g.mL^−1^ of AND-2-HyP-*β*-CYD complex. On the other hand, remedisivir was taken as standard in identical concentration range. MTT dye stock solution (0.5 mg.mL^−1^) was incorporated to each well of the microtiter plate post completion of incubation time. The plate was further incubated in 5% CO_2_ incubator at 37 °C for 4 h. The formazon crystals liberated after cell lysis, were suspended in 100 *μ*L of DMSO (Dimethyl sulfoxide) in the last step. The absorbance was captured using reference wavelength of 630 nm in a ELISA reader at 570 nm.

#### 2.6.2 Antiviral testing

The antiviral testing of solid complex was carried out using qRT-PCR assay method [25]. Briefly, 1 × 10e^4^ Vero E6 cells were suspended per well and incubated at 37 °C for 8 h. Further, Vero E6 cells were incubated with 0.02, 0.05, 0.11, 0.22, 0.45, 0.9, and 1.8 *μ*g.mL^−1^ concentration of AND-2-HyP-*β*-CYD complex. On the other hand, 1% ethanol only was incubated with control cells. The Vero E6 cells were infected with SARS-CoV-2 at MOI (Multiplicity of Infection) of 0.01. Following this, viral RNA was isolated from 100 μl culture supernatant after 48 h and this was subjected to qRT-PCR (in duplicates). In this way, Ct (Cycle Threshold Value) values for N and E gene sequence were determined. Suppression of virus replication was observed on the fold change in the Ct value in AND-2-HyP-*β*-CYD complex treated cells compared to the control. Remdesivir was employed as a standard for virus inhibition. IC_50_ values were determined using AAT Bioquest IC50 calculator.

### 2.7 Pharmacokinetic analysis

The pharmacokinetic analysis was carried out in male Sprague Dawley rats. The animals were kept in light controlled environment (Photoperiod~12 h, 23 ± 1 °C, 50 ± 5% humidity) with access to food and water *ad libitum*. The *in vivo* study was performed in agreement with the direction of Committee for the Purpose of Control and Supervision of Experiments on Animal (CPCSEA), Government of India with prior approval from Institutional Animal Ethics Committee (IAEC). In order to evaluate the potential of 2-HyP-*β*-CYD to intensify the oral bioavailability of AND, HPLC assay method was employed for measuring the concentration of AND in plasma samples as specified earlier. In brief, 120 male rats were indiscriminately divided into 30 groups of 4 animals each similar to blood collection time points. AND and AND equivalent to 100 mg.kg^−1^ in AND-2-HyP-*β*-CYD complex was administered orally by gavages. Rats were fasted (Approximately 4 h) before administration of drug. On the other hand, AND at the dose of 10 mg.kg^−1^ was administered intravenously (*i.v.*) for comparative studies.

The AND in plasma was monitored up to 48 h post oral administration of AND and AND-2-HyP-*β*-CYD complex. Blood samples were collected in both groups at 0.083, 0.25, 0.5, 1, 2, 4, 8, 16, 24 and 48 h. About 750 *μ*l of blood sample was isolated from the retro-orbital vein into microcentrifuge tubes containing 100 *μ*l of 10 % w/v sodium citrate as anticoagulant. The plasma was extracted from the blood by centrifugation at 5000 rpm for 20 min at 4 °C and stored at -40 °C until analyzed.

To extract the AND from plasma samples, plasma was typically mixed with methanol (1: 3 ratio) and vortexed for 20 min. Post mixing, the homogenate was centrifuged for 20 min at 5000 rpm to extract the proteins [26]. The supernatant was filtered through 0.22 *μ*m membrane filter and 10 *μ*l was injected as specified earlier in to the HPLC system.

Plasma concentration data were analyzed and computed for one-compartmental pharmacokinetic parameters. The following pharmacokinetic parameters were assessed: *ke* (h), *t*_1/2_ (h), C_max_ (*μ*g.mL^−1^), AUC_last_ (h.*μ*g.mL^−1^), AUC_inf_ (h.*μ*g.mL^−1^), MRT (h), CL_Total_ (L.h^−1^), Vd (L) and F %.

### 2.8 Statistical analysis

Statistical significance was examined using One-way-analysis of variance (One-Way-ANOVA) and Unpaired-*t* test. *P* < 0.05 was seized as a significant level of difference. All the observations were expressed as the average ±S.D for n ≥ 3.

## 3 Results and discussion

### 3.1 Synthesis and confirmation of AND-2-HyP-β-CYD using analytical and spectral techniques

**Figure 1:**
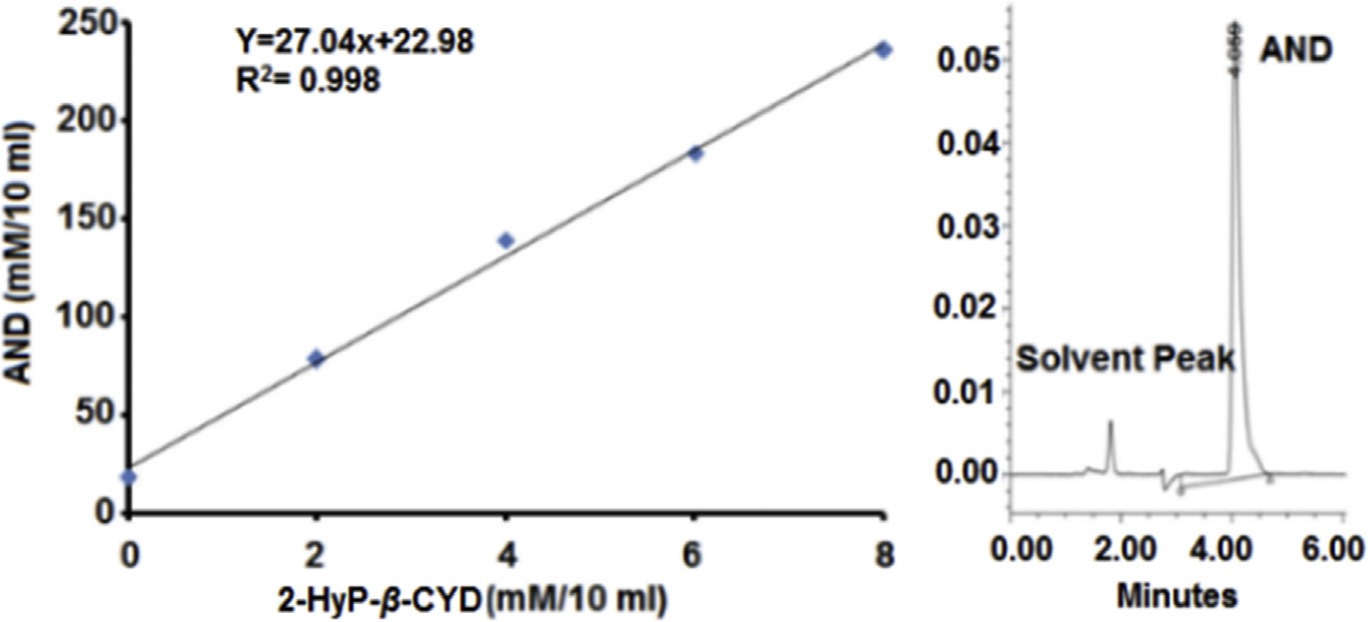
Phase solubility analysis for determining the ratio of AND-2-HyP-*β*-CYD complex in liquid phase with representative chromatogram of AND at 4.1 min.

The main focal point of the current study was to employ 2-HyP-*β*-CYD, a bio-compatible and non-toxic customized cyclic oligomers of glucose to raise the solubility and dissolution features, absolute oral bioavailability and hence, the therapeutic index of AND, an antiviral agent with a peculiar manner of action that holds sincere prospect in inhibiting the SARS-CoV-2. A molecular inclusion approach was utilized to modify and solubilize AND in the current study via the solvent evaporation technique [19]. Post synthesis of the AND-2-HyP-*β*-CYD complex, next step was to find out the arrangement, ratio, and the *K*c of the complex. Hence, phase solubility assay method was adopted to measure the stoichiometry of the complex in solution phase [27]. The phase-solubility curve of AND-2-HyP-*β*-CYD complex in solution is illustrated in Figure 1. The AND solubility was increased linearly as a function of 2-HyP-*β*-CYD concentration. This information allowed to recognize the phase-solubility curve as *A*_L_ type in AND-2-HyP-*β*-CYD complex [27]. The lineal host-guest correlativity with slope (<1) advised synthesis of a 1:1 ratio complex of AND with 2-HyP-*β*-CYD. The *k*c of AND-2-HyP-*β*-CYD complex in solution phase was calculated to be 38.60 x 10^−3^ M.

Next, AND-2-HyP-*β*-CYD complex in solid state was characterized using ATR spectroscopy. ATR spectra were captured to examine the modification of frequencies that happened due to hydrophobic interactions during the inclusion of AND in 2-HyP-*β*-CYD cavity. The chart of ATR stretching frequencies of AND, 2-HyP-*β*-CYD, physical mixture and AND-2-HyP-*β*-CYD complex are stated in Table 1. The AND spectrum demonstrated a typical peak at 1,723 cm^−1^ (ν, C=O), pointed out the existence of a lactone ring. Moreover, peaks of therapeutic entity were remarked at 1,633 cm^−1^ and 1,074 cm^−1^ that proposed presence of –C=C and -C-O bond, respectively. Other peaks detected for AND were 2,925 cm^−1^ and 2,849 owing to the various OCH_3_ and CH_3_ groups. The 2-HyP-*β*-CYD spectrum demonstrated the vibration of free –OH groups at 3,343 cm^−1^ whereas 2,927 cm^−1^ and 1,022 cm^−1^ displayed the existence of –CH_3_ stretching and C–O–C bending, respectively. The physical mixture of AND with 2-HyP-*β*-CYD pointed out the existence of monovular peaks of individual components. Finally, spectrum of AND-2-HyP-*β*-CYD complex suggested the synthesis due to the presence of intact lactone (1727 cm^−1^) and –C=C- bond (1673 cm^−1^) of drug in addition to 2925 cm^−1^ (-CH_2_/CH_3_) and 3338 cm^−1^ (-OH group) of 2-HyP-*β*-CYD (Table 1).

**Table 1:** Attenuated Total Reflectance (ATR) analysis of AND, 2-HyP-*β*-CYD, physical mixture of AND and 2-HyP-*β*-CYD (1:1) and AND-2-HyP-*β*-CYD complex prepared by solvent evaporation method.

Sample	Peaks (cm^−1^)	Assignment
AND	3393	Intermolecular Hydrogen bonding
	2925	-CH_2_/-CH_3_
	2849	-CH_2_/-CH_3_
	1723	Sharp peak (Lactone ring)
	1673	-C=C- bond
	1074	-C-O
	1031	-C-O
2-HyP-*β*-CYD	3343	-OH (stretching)
	2927	-CH_3_/-CH_2_ (Stretching)
	1022	-C-O-C (bending)
	1079	-C-O stretching
Physical Mixture (1:1)	3256	-OH group
	1031	-C-O
	1070	-C-O
AND-2-HyP-*β*-CYD complex	3338	-OH group
	2925	-CH_3_/-CH_2_
	1727	(Lactone ring)
	1673	(-C=C-) bond
	1079	-C-O stretching
	1024	-C-O

Note: AND: Andrographolide; 2-HyP-*β*-CYD: 2-Hydroxypropyl-*β*-cyclodextrin; AND-2-HyP-*β*-CYD complex.

**Figure 2:**
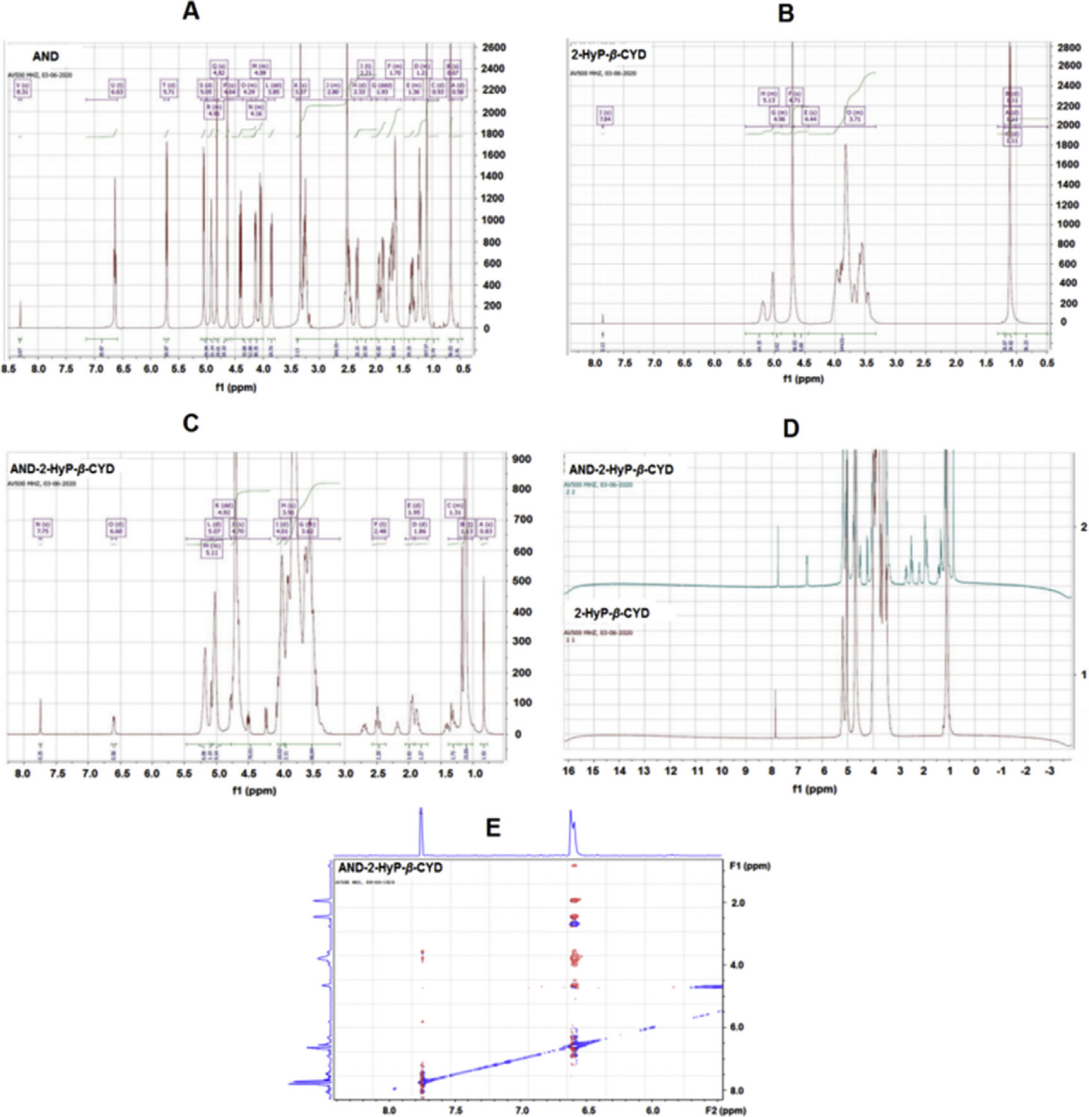
^1^H NMR spectroscopy of (A) AND in DMSO-d_6_, (B) 2-HyP-*β*-CYD in D_2_O, (C) AND-2-HyP-*β*-CYD in D_2_O, (D) Comparison between 2-HyP-*β*-CYD and AND-2-HyP-*β*-CYD and (E) 2 D NMR (ROESY) spectroscopy of AND-2-HyP-*β*-CYD in D_2_O.

The complex in solid state was also ascertained using ^1^H NMR spectroscopy. The spectra informed about chemical shift variations in free and bound state of therapeutic entity. The consequent Δ*δ* (chemical shift), is depicted as change between bound and free therapeutic molecule chemical shifts. Such consequent chemical shifts were estimated by employing the equation Δ*δ* = *δ*_complex_-*δ*_free_ [28]. Ultimately, this formula yielded downfield-and upfield chemical shifts, respectively. The spectrum of AND, 2-HyP-*β*-CYD and AND-2-HyP-*β*-CYD is shown in Figure 2. The H3 as well as H_5_ protons (3.71 ppm) situated in the vicinity of 2-HyP-*β*-CYD were shifted upfield (3.62 ppm) due to molecular interaction with AND, betraying the synthesis of solid complex by the inclusion mode. In addition, the shift in the signals for the protons H_1_ and H_2_ in addition to H_4_, and H_6_ active on the surface of 2-HyP-*β*-CYD informed about the host molecule's conformational alteration in the presence of therapeutic entity. ^1^H NMR spectrum of AND indicated the presence of triplet at 6.63 ppm (Figure 2) which was up-fielded in AND-2-HyP-*β*-CYD complex at 6.60 ppm (doublet) confirmed the introduction of AND in cavity of 2-HyP-*β*-CYD through lactone ring. This is in agreement with the ATR data. Moreover, intermolecular interactions in AND-2-HyP-*β*-CYD complex were confirmed by 2D ROESY spectroscopy [29] and presented in Figure 2. 2D NMR spectroscopy is a vital technique for the characterization of solid complex in solution state. This illustrates the existence of cross-peaks between protons from both CYD and guest molecule. In addition, the comparative intensities of cross-peaks depend on the spacing between the related protons. ROESY also correlates the triplet at 6.63 ppm of AND with H-3 protons (3.71 ppm) of 2-HyP-*β*-CYD (Figure 2).

**Figure 3:**
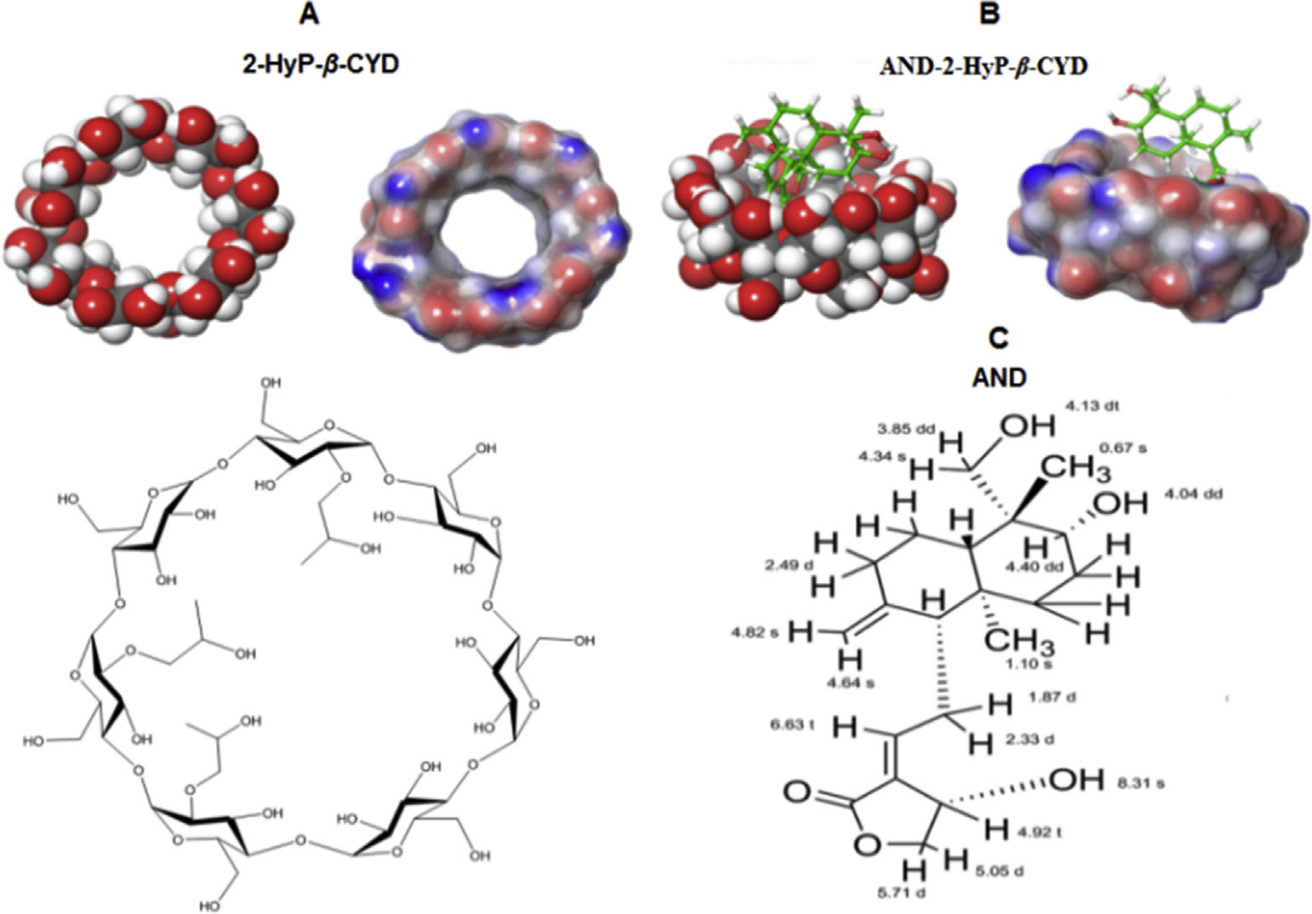
(A) Schematic representation of molecular and chemical structure of 2-HyP-*β*-CYD (B) Conformation molecular modeling structures of AND-2-HyP-*β*-CYD complex and (C) Chemical structure of 2-HyP-*β*-CYD.

Further, *in silico* docking study was carried out to pretend the orientation of stable chemical structure of AND-2-HyP-*β*-CYD complex. The docking studies revealed that the 2-hydroxy-ethylidene furanone part of AND is posing into the cavity of 2-HyP-*β*-CYD. The docked orientation of the AND displayed a sequence of electrostatic and hydrophobic non-bonded interactions that have stabilized the AND-2-HyP-*β*-CYD complex. On the other hand, hexahydro naphthalenyl segment of AND was remained outside the 2-HyP-*β*-CYD cavity. The binding affinity exhibited in terms of glide docking score for the interaction of AND with 2-HyP-*β*-CYD was measured to be -5.026 kcal.mol^−1^ (Table 2). It was observed that AND was best docked to 2-HyP-*β*-CYD with Glide energy of -29.807 kcal.mol^−1^, potential energy of -3535.08 kcal.mol^−1^, Van der Waals energy of -6797.27 kcal.mol^−1^ and electrostatic energy of -4060.78 kcal.mol^−1^ (Figure 3 and Table 2). Further, share from Van der Waals energy was higher in comparison to electrostatic energy for AND-2-HyP-*β*-CYD complex stabilization as indicated in Table 2.

**Table 2:** Parameters for the AND and 2-HyP-*β*-CYD complex calculated using force field OPLS3.

Parameters	Score kcal/mol	Glide Parameters	Score *k*cal/mol
Potential energy	-3535.08	Glide energy	-29.807
Stretch energy	301.838	Glide Ligand efficiency, sa	-0.201
Bend energy	1275.981	Glide Ligand efficiency, In	-1.191
Dihedral energy	1156.933	Glide gScore	-5.026
Electrostatic energy	-4060.78	Glide docking Score	-5.026
Van der Waals energy	-6797.27	Glide emodel	-37.919

**Figure 4:**
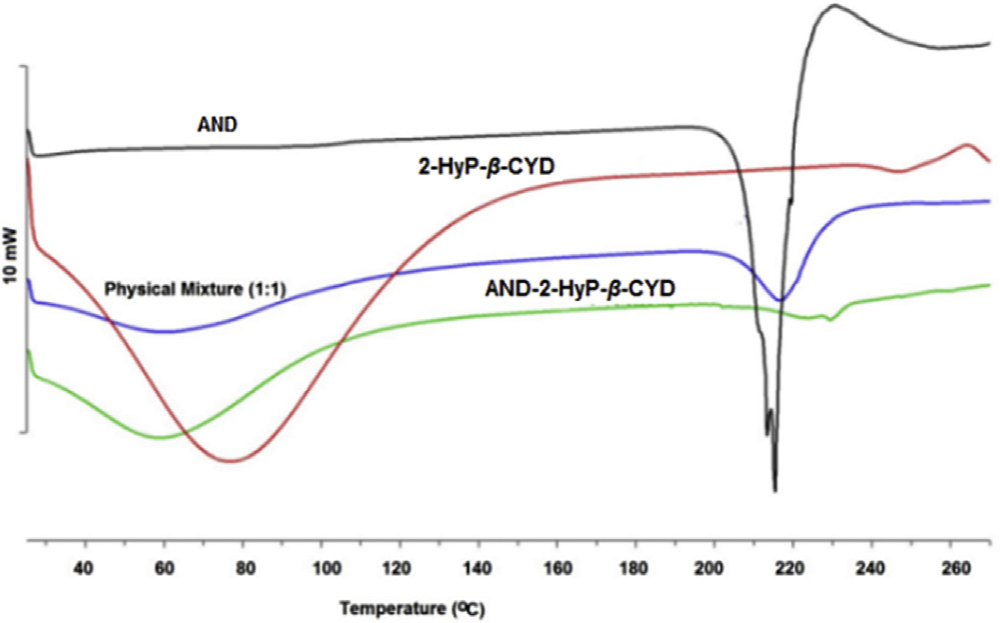
DSC of AND, 2-HyP-*β*-CYD, physical mixture of AND and 2-HyP-*β*-CYD and AND-2-HyP-*β*-CYD complex carried out between 30-260 °C (heating rate of 10 °C/min) using N_2_ as inert gas.

In addition, AND-2-HyP-*β*-CYD complex in solid state was also characterized using DSC to notice the endothermic peak and comparison was made with respective components as presented in Figure 4. Thermogram demonstrated that the endothermic peak of AND was noticed at 214.81 °C close to the melting point range (225–226 °C). The thermograms of all cyclodextrins owing to dehydration process (CDs i.e. *α*-, *β* and *γ*-CDs) display a wide range of endothermic peaks ranging from 40 to 150 °C (77.52 °C for 2-HyP-*β*-CYD). The thermogram of the physical mixture of AND with 2-HyP-*β*-CYD depicted the presence of identical peaks of individual components. However, thermogram of AND-2-HyP-*β*-CYD complex displayed a complete absence of characteristic endothermic peak of AND and a new peak of AND-2-HyP-*β*-CYD complex was appeared at 60.40 °C.

**Figure 5:**
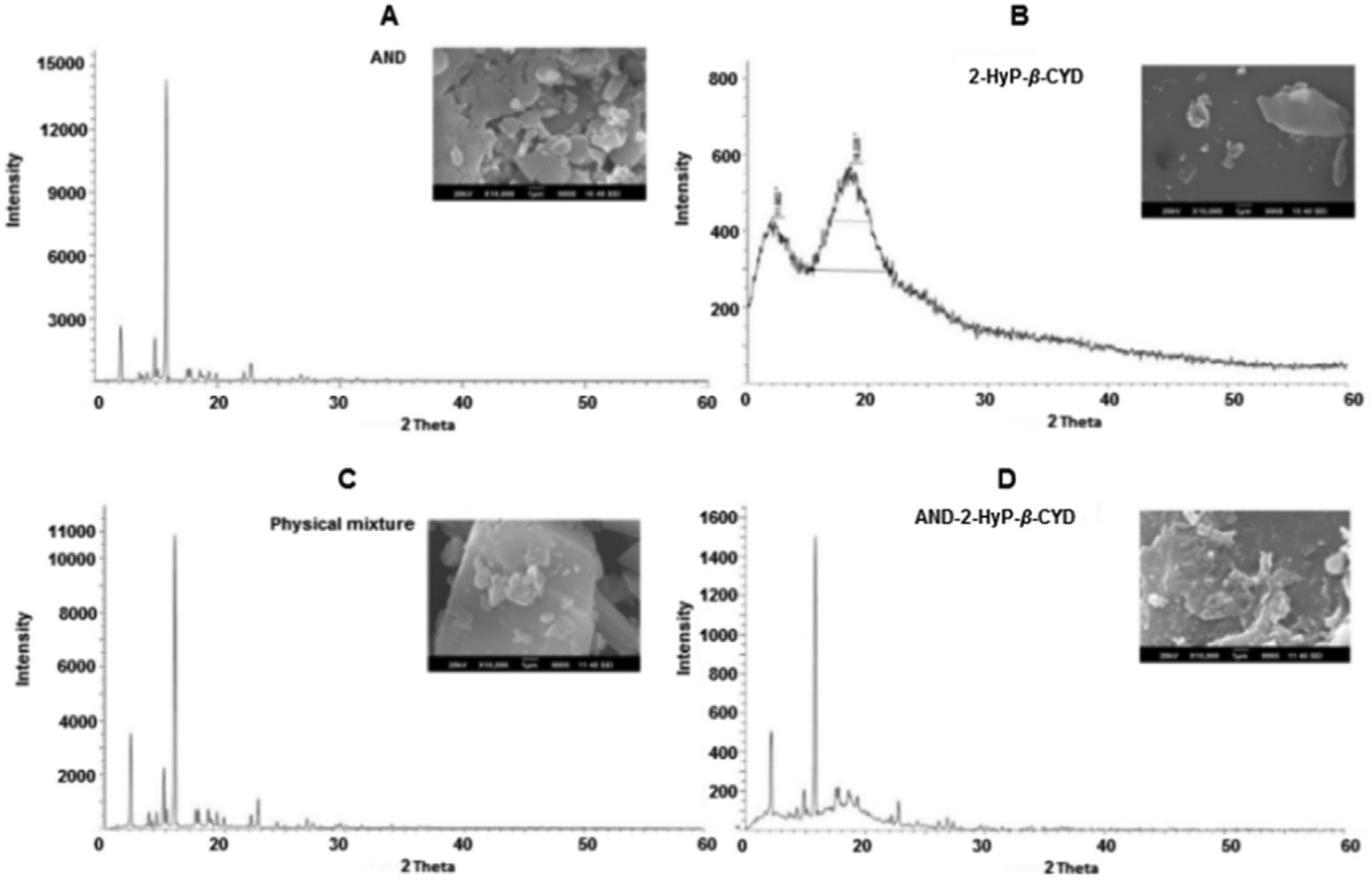
PXRD pattern of (A) AND, (B) 2-HyP-*β*-CYD, (C) Physical mixture of AND and 2-HyP-*β*-CYD and (D) AND-2-HyP-*β*-CYD complex at 2*θ* between 0-60° in addition to corresponding scanning electron microscopy photographs at the scale of 1 *μ*m.

**Figure 6:**
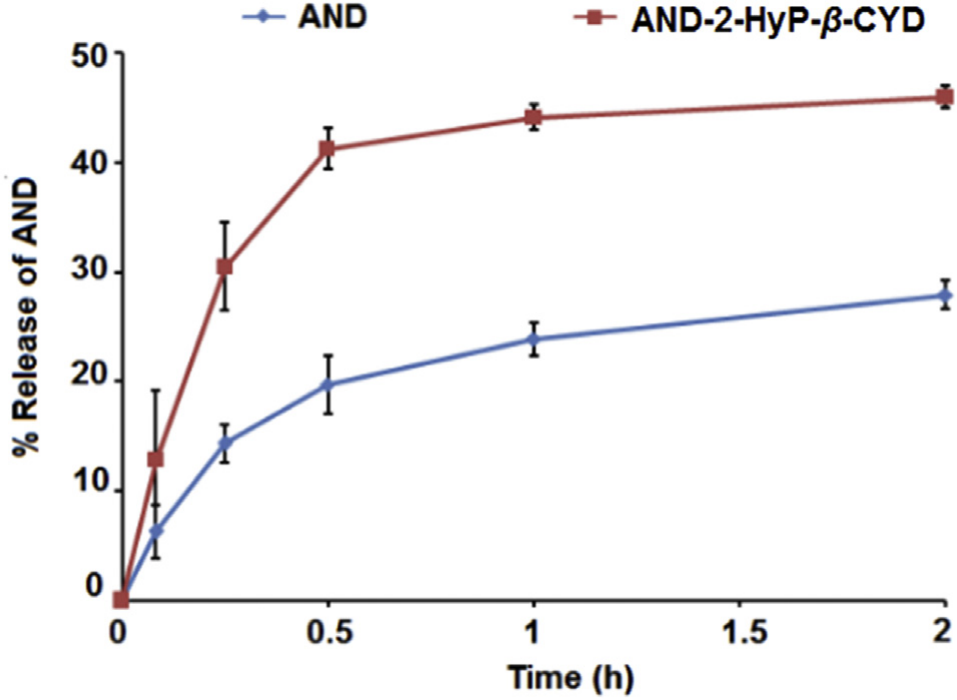
Dissolution testing of AND and AND-2-HyP-*β*-CYD complex in SIF (pH~ 7.2). AND-2-HyP-*β*-CYD complex displayed significantly (*P* < 0.05) higher release of 45.91 ± 0.99% of therapeutic entity in comparison to 27.89 ± 1.32% of AND at the identical time interval.

PXRD was utilized to designate the crystal geometry of the solid complex. The diffractogram of AND displayed pointed peaks showing its crystalline geometry (Figure 5). The 2-HyP-*β*-CYD demonstrated vague, wide and distributed peaks. Nevertheless, the physical mixture demonstrated few sharp and diffused peaks with devalued strengths. Although this stands for reduction of crystalline structure, however; a few sharp peaks were also noticed signaling the impression of AND. Eventually, PXRD of AND-2-HyP-*β*-CYD solid complex illustrated vague, broad diffused peaks of vitiated intensities with limited sharp peaks. This confirmed the significant transformation of AND crystalline architecture in to amorphous geometry in AND-2-HyP-*β*-CYD solid complex. Next, SEM was exploited to figure out the microscopical aspects of therapeutic entity before and after complexation. Photomicrograph of AND informed the presence of crystalline stuff of asymmetrical size (Figure 5). 2-HyP-*β*-CYD also seemed as particles with no decisive structure. Furthermore, physical mixture highlighted the crystalline geometry of both AND and 2-HyP-*β*-CYD instead of AND-2-HyP-*β*-CYD complex which displayed small size particles aiding to aggregation, recommended the creation of an amorphous structure with the presence of a single component in the complex, and thus pointed out the absolute complexation. The assay of AND-2-HyP-*β*-CYD complex informed the existence of 17.03 ± 0.60 % of AND within compliance. AND-2-HyP-*β*-CYD solid complex displayed the solubility of 17.15 ± 1.52 *μ*g.mL^−1^ significantly (Unpaired t test, *P* < 0.05) higher than 6.39 ± 0.47 *μ*g.mL^−1^ of AND in the aqueous phase. This depicted a 2.68 fold increment in the aqueous solubility of AND upon complexation. The dissolution study of AND and AND-2-HyP-*β*-CYD solid complex was carried out in SIF (pH 7.2) (Figure 6). AND demonstrated 27.89 ± 1.32 % release from hard gelatin capsule after 2 h, instead of AND-2-HyP-*β*-CYD complex displayed significantly (*P* < 0.05) higher release (45.91 ± 0.99 %) at the identical time interval. Recently, US FDA, has placed more stress on dissolution chart of therapeutic moieties. A dissolution chart can help to qualify the product more accurately than a single point dissolution test under suitable test conditions. The F1, factor is relative to the mean difference between the two profiles, whereas F2 factor is reciprocally proportional to the mean squared difference between the two charts, with emphasis on the larger deviation among all the time points. The F2 factor depicts the closeness between the two charts. During the comparison of dissolution profile, when the two charts are similar, then F 2 is equal to 100. The F 2 value was measured to be 40 % indicating no similarity between dissolution profile of AND and AND-2-HyP-*β*-CYD solid complex.

### 3.2 AND-2-HyP-β-CYD complex offered superior antiviral activity against SARS-CoV-2 infected in Vero6 cells

The AND-2-HyP-*β*-CYD complex did not show any cytotoxicity to Vero E6 cells at 10 *μ*g.mL^−1^ or 100 *μ*g.mL^−1^, measured by MTT assay [24]. AND-2-HyP-*β*-CYD complex at the concentration of 9 mg.mL^−1^ demonstrated 85.97 % (E) and 85.89 % (N) inhibition of Vero E6 cells infected with SARS-CoV-2 at 24 h remarkably (One-Way-ANOVA test, P < 0.05) higher than 72.26 % (E) and 77.61 % (N) presented by standard drug, remdesivir (Table 3). Correspondingly, AND-2-HyP-*β*-CYD complex at the concentration of 0.9 mg.mL^−1^ demonstrated 86.38% (E) and 85.95% (N) inhibition of Vero E6 cells infected with SARS-CoV-2 at 24 h remarkably (One-Way-ANOVA test, P < 0.05) greater than 72.26% (E) and 77.61% (N) presented by standard drug, remdesivir. On the other hand, there was insignificant (One-way-ANOVA test, P > 0.05) deviation in antiviral efficacy of AND-2-HyP-*β*-CYD complex at 9 mg.mL^−1^ or 0.9 mg.mL^−1^ in comparison to remdesivir at 48 h. Remdesivir displayed 0.09 *μ*g.mL^−1^ as IC_50_ for E gene and 0.06 *μ*g.mL^−1^ for N gene importantly (One-way-ANOVA test, P < 0.05) lesser than 0.1 *μ*g.mL^−1^ (E gene) and 0.29 *μ*g.mL^−1^ (N gene) exhibited by AND-2-HyP-*β*-CYD complex. Remdesivir or triphosphate form of remdesivir, a nucleotide analogue, is utilized as a substrate for some RNA-dependent RNA-polymerase (RdRp) of virus. Further, remdesivir suppresses the synthesis of viral RNA through a particular phenomena of delayed chain termination in three variants (MERS-CoV, SARS-CoV and SARS-CoV-2) till noted of coronaviruses [30]. On the other hand, AND inhibits a specific region of the protease of SARS-CoV-2 virus [6]. Hence, it may be speculated that owing to distinct and accurate mechanism of action, remdesivir exhibited remarkably lower IC_50_ value in comparison to AND.

**Table 3:** Percent cell viability and inhibition of virus replication displayed by standard antiviral drug remdesivir and AND-2-HyP-*β*-CYD compound.

Sample	% Cell viability against Vero E6 cells		% Inhibition of virus replication post infection (Vero E6 cells infected with SARS-CoV-2)			
	24 h	48 h	24 h		48 h	
			E	N	E	N
Remdesivir (Standard Antiviral drug)	99.23	94.37	72.26	77.61	99.64	99.76
AND-2-HyP-*β*-CYD Complex (9 mg/ml)	95.45	92.16	85.97	85.89	98.3	97.7
AND-2-HyP-*β*-CYD Complex (0.9 mg/ml)	80.27	54.97	86.38	85.95	99.90	99.91

**Note**: AND-2-HyP-*β*-CYD: Andrographolide-2-Hydroxypropyl-*β*-Cyclodextrin complex. AND-2-HyP-*β*-CYD complex at the concentration of 9 mg.mL^−1^ demonstrated 85.97% (E) and 85.89% (N) inhibition of Vero E6 cells infected with SARS-CoV-2 at 24 h remarkably (One-Way-ANOVA test, P < 0.05) higher than 72.26% (E) and 77.61% (N) presented by standard drug, remdesivir. AND-2-HyP-*β*-CYD complex at the concentration of 0.9 mg.mL^−1^ demonstrated 86.38% (E) and 85.95% (N) inhibition of Vero E6 cells infected with SARS-CoV-2 at 24 h remarkably (One-Way-ANOVA test, P < 0.05) greater than 72.26% (E) and 77.61% (N) presented by standard drug, remdesivir.

### 3.3 AND-2-HyP-β-CYD complex tendered augmented pharmacokinetic profile in comparison to AND only

**Figure 7:**
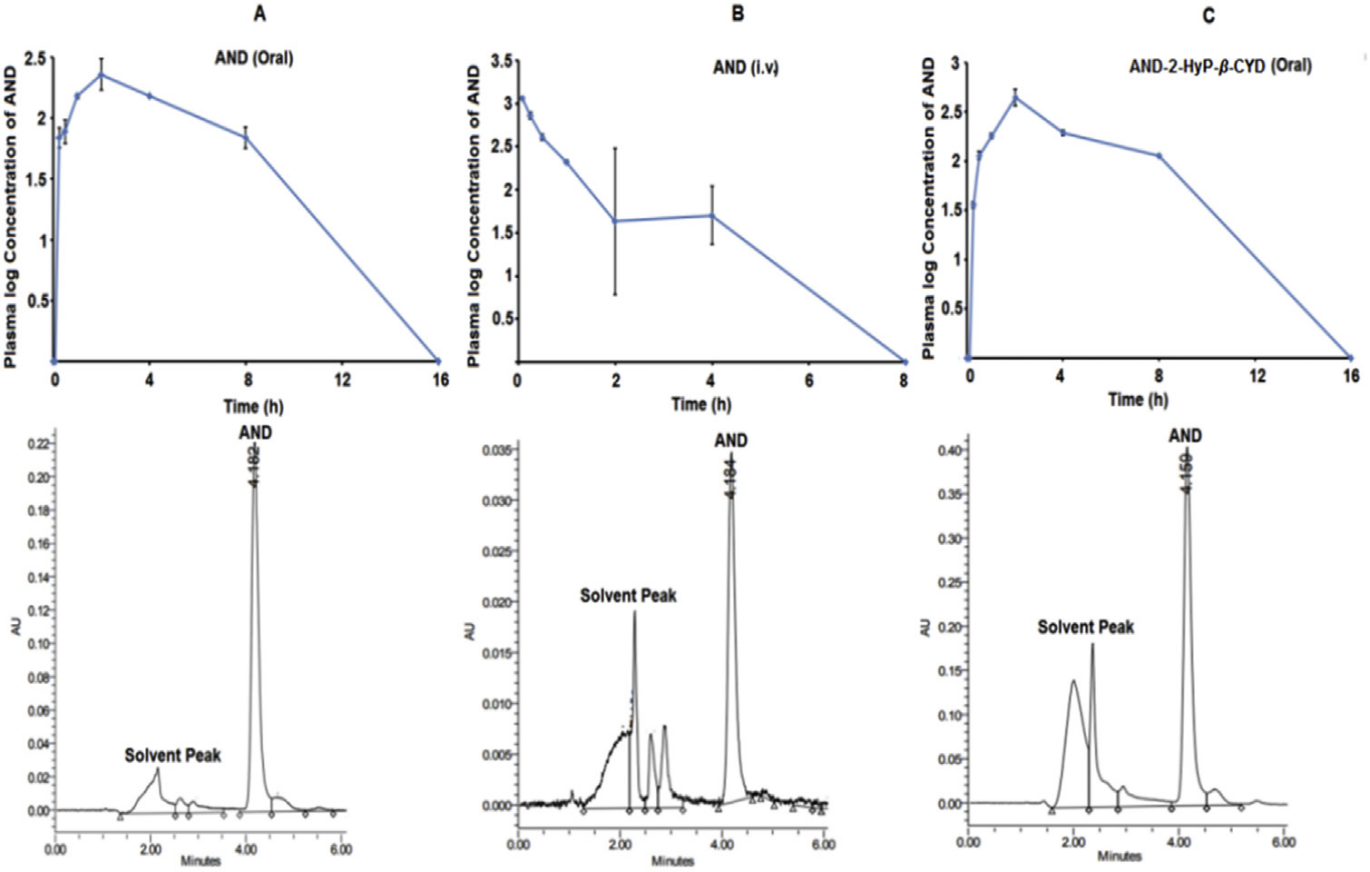
Plasma concentration-time profile curve of (A) AND at the dose of 100 mg.kg^−1^ (oral) and (B) AND at 10 mg.kg^−1^ (intravenous) administration in addition to (C) oral administration of AND-2-HyP-*β*-CYD complex equivalent to 100 mg.kg^−1^ of AND in rats with representative chromatogram.

The log plasma concentration-time chart of AND and AND-2-HyP-*β*-CYD complex was calculated and presented in Table 4 and Figure 7. AND exhibited C_max_ of 239.6 ± 75.37 *μ*g.mL^−1^ with t_max_ of 2 h at the dose of 100 mg.kg^−1^ post oral administration in rats that was remarkably (Unpaired t test, *P* < 0.05) improved (461.105 ± 103.00 *μ*g.mL^−1^) in AND-2-HyP-*β*-CYD complex indicating the enhanced absorption of drug (Table 4 and Figure 7). However, the biological half-life of AND (5.92 ± 0.21 h) was slightly altered to higher value in AND-2-HyP-*β*-CYD solid complex (6.38 ± 0.055 h). In addition, AND-2-HyP-*β*-CYD complex substantiated the improved absorption in terms of AUC_last_ of 1842.115 ± 125.51 h.*μ*g.mL^−1^ as compared (Unpaired t test, P < 0.05) to 1223.6 ± 143.09 h.*μ*g.mL^−1^ of AND. The bioavailability (F %) was computed and noticed to be 15.87 ± 3.84 % and 23.84 ± 5.46 % for AND and AND-2-HyP-*β*-CYD complex, respectively.

**Table 4:** Pharmacokinetic analysis of AND and AND-2-HyP-*β*-CYD Complex.

Parameters	AND (Oral, 100 mg/kg)	AND (i.v, 10 mg/kg)	AND-2-HyP-*β*-CYD Complex (Oral, 100 mg/kg)
*K*e (h)	0.117 ± 0.004	0.807 ± 0.02	0.1085 ± 0.001
t_*1/2*_ (h)	5.92 ± 0.21	0.859 ± 0.031	6.38 ± 0.055
C_max_ (*μ*g/ml)	239.6 ± 75.37	NA	461.105 ± 103.00
AUC_last_ (h.*μ*g/ml)	1223.6 ± 143.09	801.14 ± 181.07	1842.115 ± 125.51
AUC_inf_ (h.*μ*g/ml)	1845.58 ± 305.77	891.08 ± 179.75	2937.37 ± 108.10
MRT (h)	1.49 ± 0.0.085	1.11 ± 0.04	1.59 ± 0.05
CL_Total_ (L/h)	0.055 ± 0.008	0.011 ± 0.002	0.032 ± 0.002
Vd (L)	0.468 ± 0.060	0.013 ± 0.003	0.299 ± 0.019
∗F (%)	15.87 ± 3.84	NA	23.84 ± 5.46%

All measurements were carried out in quartet (n = 4). ∗Bioavailability values are subject to age, gender, species variations etc. Difference ≥25% intra-batch or batch-to-batch variability in bioavailability necessitates *in vivo* tests for batch certification. Any difference in bioavailability is likely to be less than 10%. *Ke*: Elimination rate constant; t_1/2_: Half life; C_max:_ Maximum concentration; AUC_last_: Area Under the curve; AUC_inf_: Area under the curve up to infinite; MRT: Mean Residence Time; CL_Total_: Total Body Clearance; V_d_: Volume of Distribution: F (%): Bioavailability. AND exhibited C_max_ of 239.6 ± 75.37 *μ*g.mL^−1^ with t_max_ of 2 h at the dose of 100 mg.kg^−1^ post oral administration in rats that was remarkably (Unpaired t test, *P* < 0.05) improved (461.105 ± 103.00 *μ*g.mL^−1^) in AND-2-HyP-*β*-CYD complex. AND-2-HyP-*β*-CYD complex substantiated the improved absorption in terms of AUC_last_ of 1842.115 ± 125.51 h.*μ*g.mL^−1^ as compared (Unpaired t test, P < 0.05) to 1223.6 ± 143.09 h.*μ*g.mL^−1^ of AND.

## 4 Conclusion

The solubility and bioavailability of AND may be increased by fabricating the 1:1 mM complex with 2-HyP-*β*-CYD. Moreover, the present investigation exhibits that AND-2-HyP-*β*-CYD complex may efficaciously change the fraction of unionized therapeutic entity in the SIF which further heightens the extent of absorption and absolute oral bioavailability of therapeutic entity. Thence, the current investigation supported that AND-2-HyP-*β*-CYD (1:1 mM) complex may be employed for scheming antiviral dosage forms to control the current COVID-19 pandemic; however, it should be studied under the framework of rigorous *in vitro* and *in vivo* parameters for clinical translation.

## Declarations

### Author contribution statement

Shashi Chandrama Singh: Conceived and designed the experiments; Performed the experiments; Analyzed and interpreted the data; Contributed reagents, materials, analysis tools or data; Wrote the paper.

Dharmendra Kumar Khatri: Conceived and designed the experiments; Performed the experiments; Analyzed and interpreted the data.

Kulbhaskar Singh; Vinay Kumar Kanchupalli: Conceived and designed the experiments.

Jitender Madan: Analyzed and interpreted the data.

Shashi Bala Singh; Harshpal Singh: Contributed reagents, materials, analysis tools or data.

### Funding statement

This work was supported by M/s Ambe Phytoextracts Private Limited, Pauri Garhwal, Uttarakhand, India.

### Data availability statement

Data will be made available on request.

### Declaration of interests statement

The authors declare no conflict of interest.

### Additional information

No additional information is available for this paper.
